# Cell cycle inhibitors protect motor neurons in an organoid model of Spinal Muscular Atrophy

**DOI:** 10.1038/s41419-018-1081-0

**Published:** 2018-10-27

**Authors:** Jin Hui Hor, Eunice Shi-Yi Soh, Li Yi Tan, Valerie Jing Wen Lim, Munirah Mohamad Santosa, Winanto, Beatrice Xuan Ho, Yong Fan, Boon-Seng Soh, Shi-Yan Ng

**Affiliations:** 1 Institute of Molecular and Cell Biology, 61 Biopolis Drive, Singapore, 138673 Singapore; 2 Department of Biological Sciences, National University of Singapore, 14 Science Drive 4, Singapore, 117543 Singapore; 3 School of Biological Science, Nanyang Technological University, Singapore, 637551 Singapore; 4 The Third Affiliated Hospital of Guangzhou Medical University, 510150 Guangzhou, China; 5 National Neuroscience Institute, 11 Jalan Tan Tock Seng, Singapore, 308433 Singapore; 6 Department of Physiology, National University of Singapore, 28 Medical Drive, Singapore, 117456 Singapore

## Abstract

Spinal Muscular Atrophy (SMA) is caused by genetic mutations in the *SMN1* gene, resulting in drastically reduced levels of Survival of Motor Neuron (SMN) protein. Although SMN is ubiquitously expressed, spinal motor neurons are one of the most affected cell types. Previous studies have identified pathways uniquely activated in SMA motor neurons, including a hyperactivated ER stress pathway, neuronal hyperexcitability, and defective spliceosomes. To investigate why motor neurons are more affected than other neural types, we developed a spinal organoid model of SMA. We demonstrate overt motor neuron degeneration in SMA spinal organoids, and this degeneration can be prevented using a small molecule inhibitor of CDK4/6, indicating that spinal organoids are an ideal platform for therapeutic discovery.

## Introduction

Spinal Muscular Atrophy (SMA) is the most common form of motor neuron disease affecting children. It is a genetic disease caused by homozygous mutations or deletions in the SMN1 gene, resulting in drastically reduced amounts of the SMN protein. SMA manifests clinically as a childhood motor neuron disease, with the death of spinal motor neurons and subsequent denervation of skeletal muscles resulting in arrested childhood developmental milestones, paralysis and eventually death in severe SMA. The SMN2 gene in humans primarily gives rise to truncated and partially functional protein lacking exon 7, known as SMNΔ7. As such, copy number variation in the SMN2 gene is known to affect clinical severity of SMA patients. SMA is classified into four categories (SMA Type I to Type IV), with Type I as the most severe and Type IV being adult-onset. While most Type I patients have between 1 and 2 copies of SMN2, Type IV patients can have between 4 and 6 copies of SMN2^1^.

Although SMN is ubiquitously expressed, it is still not completely understood why motor neurons are one of the most severely affected cell types. The roles of SMN have not been exhaustively characterized, but it is best known as a component of the spliceosome, and widespread splicing defects have been reported in SMA and SMN-deficient cultures^2–4^. Due to its importance as a splicing regulator and the observation that SMN-null mice are embryonic lethal^5^, it has been suggested that SMA is also a neurodevelopmental disorder, where motor neurons in the spinal cord do not properly form, and those that eventually survive would rapidly degenerate postnatally. To evaluate the neurodevelopmental defects in SMA, we derived spinal organoids from patient induced pluripotent stem cells (iPSCs) and found that neurodevelopment was not significantly altered. We also report that spinal organoids are a good platform for testing small molecules that promote motor neuron survival.

## Results

### Derivation of spinal organoids from pluripotent stem cells

**Fig. 1:**
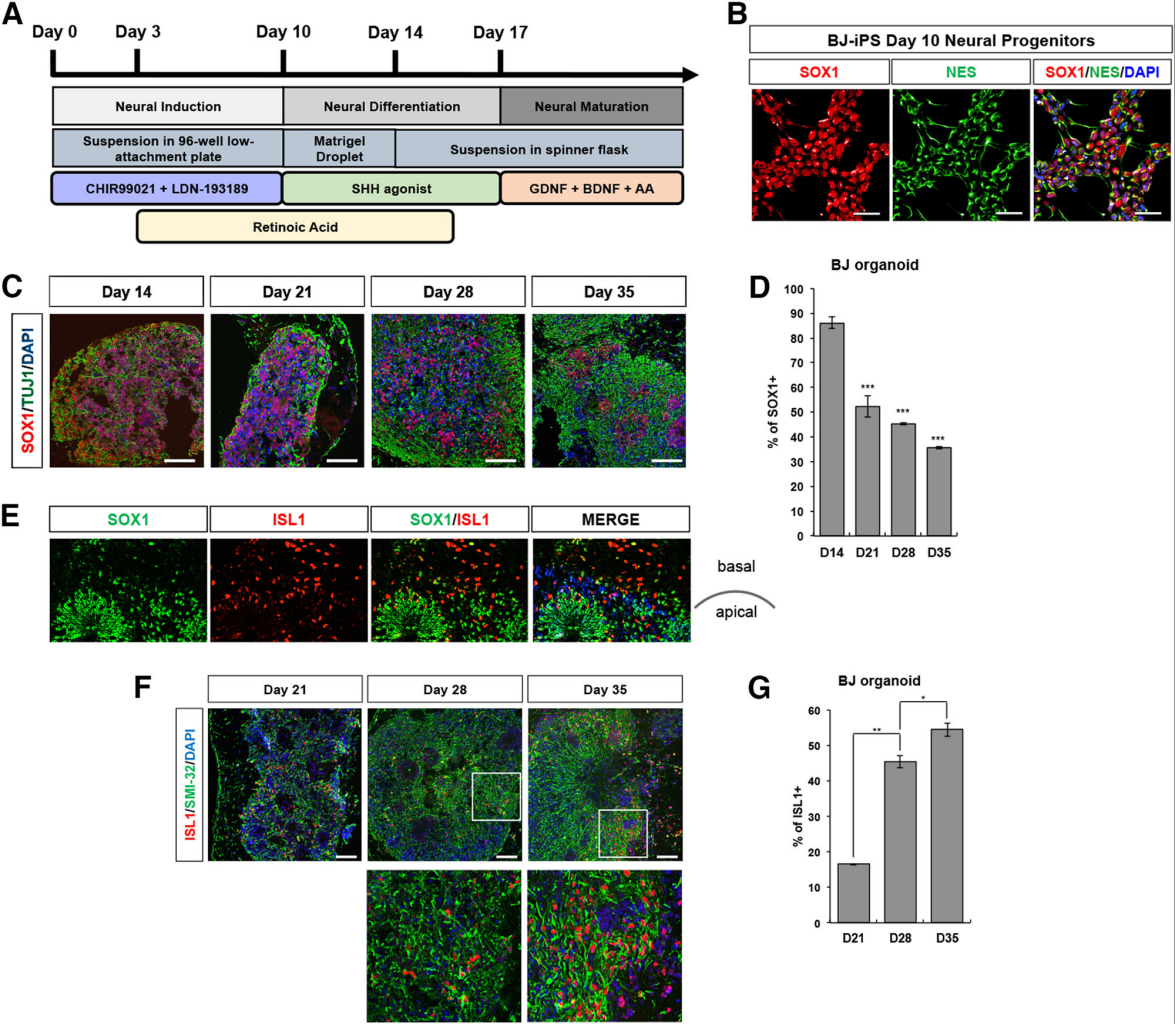
Generation of three-dimensional spinal organoids from human iPSCs. **a** Schematic illustration of spinal organoids differentiation from iPSC. **b** Co-staining of SOX1 (red) and Nestin (green) illustrating successful generation of neural progenitors in BJ-iPS motor neuron cultures. Cellular nuclei were counterstained with DAPI. Scale bars, 50 μm. **c** Representative images BJ-iPS spinal organoids at respective time points stained with SOX1 (red) and TUJ1 (green). Cellular nuclei were counterstained with DAPI. Scale bars, 100 μm. **d** Quantification of SOX1^+^ levels percentage of BJ-iPS spinal organoids at respective time points relative to total cell number. **e** Representative images of BJ-iPS spinal organoids demonstrating SOX1^+^ (green) and ISL1^+^ (red) in an apical-to-basal patterning. Cellular nuclei were counterstained with DAPI. Scale bars, 100 μm. **f** Representative images of BJ-iPS spinal organoids at respective time points stained with ISL1 (red) and SMI-32 (green). Cellular nuclei were counterstained with DAPI. Scale bars, 100 μm. **g** Graph shows percentage of ISL1^+^ at day 21, 28, and 35 in BJ-iPS spinal organoids relative to total cell number. *p < 0.05, **p < 0.01, ***p < 0.001

To generate spinal organoids, we first dissociated iPSCs into single cells, seeded 30,000 cells per well in a 96-well low-attachment plate (Supplementary Figure S1), and induced neuralization of iPSCs by blocking Bone Morphogenic Protein (BMP) signaling by LDN-193189 treatment while simultaneously activating Wnt pathways with CHIR99021 treatment^6,7^. Retinoic acid (RA) treatment begun at day 3 to caudalize the cultures, while Purmorphamine, a Sonic Hedgehog pathway agonist, was used as a ventralizing signal from days 10 to 17 (Fig. 1a). To ensure that neutralization was successful, we seeded some cells on Matrigel-coated plates, performed immunostaining on day 10 cultures and observed that cultures were homogeneously expressing neuroepithelial stem cell markers SOX1 and Nestin (Fig. 1b). At day 10, we encapsulated cells in each well with Matrigel. These were allowed to grow as stationary cultures until day 14, where the cell-Matrigel droplets were transferred into spinner flasks. To promote neuronal maturation, organoids were cultured in media supplemented with neurotrophic factors from day 17 onwards (Fig. 1a). To investigate the cellular composition and cytoarchitecture of the spinal organoids, we performed cryosectioning and immunostaining of organoids at days 14, 21, 28, and 35. At day 14, 86% of the cells were expressing SOX1, demonstrating homogeneity within the spinal organoid (Fig. 1c, d). As the spinal organoids continues to mature, SOX1^+^ cells organized into rosette structures by day 21 and continue to be present in day 28 and 35 spinal organoids (Fig. 1c). We observed a typical apical-to-basal patterning of the organoids where the apical region is marked by a layer of proliferative SOX1^+^ cells while ISL1^+^ motor neurons are present at the basal region (Fig. 1e). As differentiation proceeded, reduced number of SOX1^+^ cells were observed with the simultaneous appearance of ISL1^+^ motor neurons at day 21, showing maturation of the spinal organoids (Fig. 1f, g). ISL1^+^ motor neurons continue to rise in day 28 and 35 spinal organoids. TUJ1^+^ can also be observed to be appearing at day 14 of the spinal organoids and continue to persist in day 21, 28, and 35 spinal organoids (Fig. 1c). Together, the results demonstrate that spinal organoids are able to recapitulate spinal cord neurogenesis.

### Diverse spinal cord cell types observed in spinal organoids

**Fig. 2:**
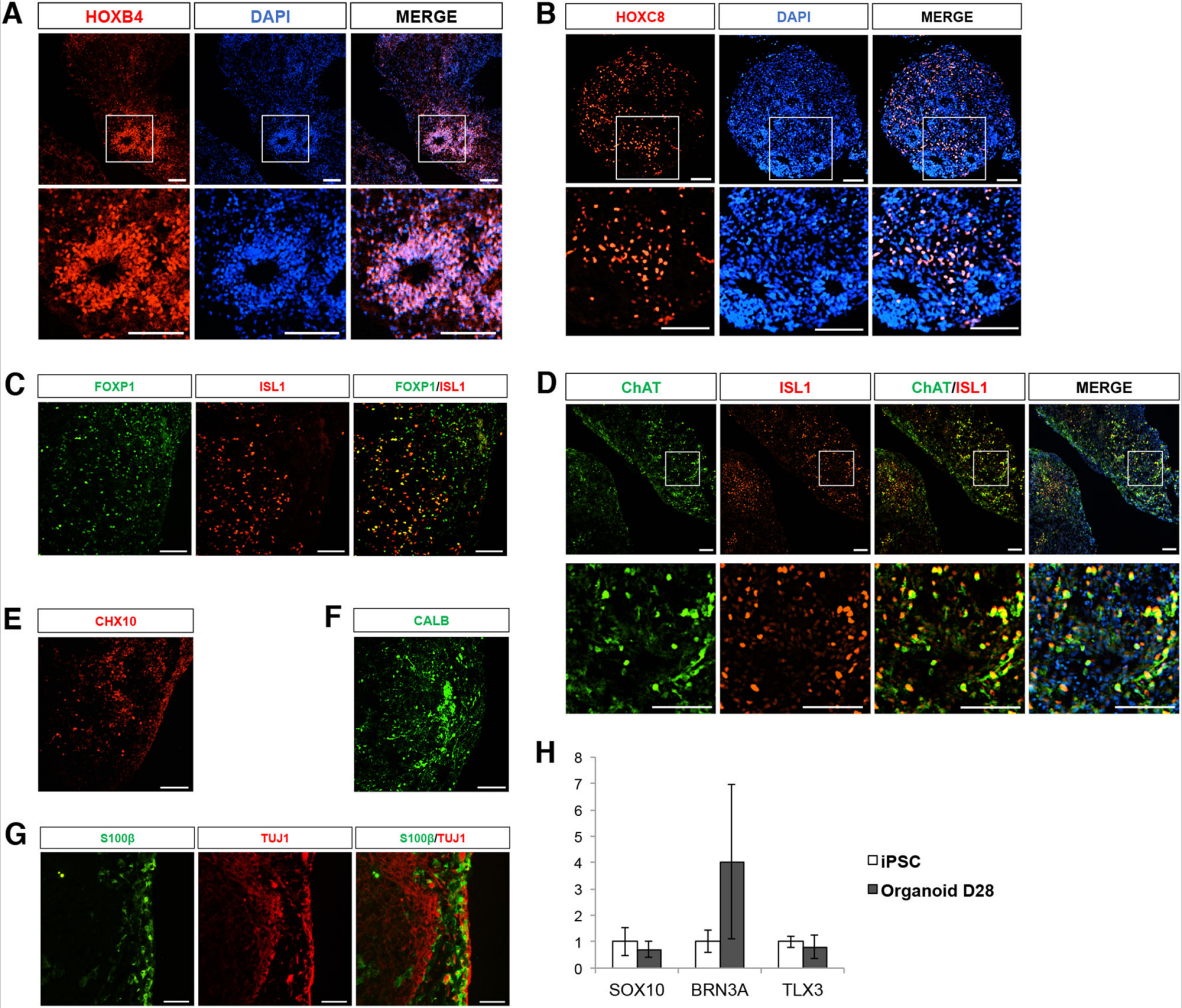
Spinal organoids consists of various spinal cord cell types. Representative images illustrating the presence of **a** HOXB4^+^ and **b** HOXC8^+^ cells in spinal organoids. Cellular nuclei were counterstained with DAPI. Scale bars, 100 μm. **c** Co-staining of FOXP1 (green) and ISL1 (red) demonstrates presence of limb-innervating neurons in spinal organoids. Scale bars, 100 μm. **d** Representative images of spinal organoids at day 42 stained with ISL1 (red) and ChAT (green). Cellular nuclei were counterstained with DAPI. Scale bars, 100 μm. Spinal organoids are stained with **e** CHX10^+^ cells (RED) and **f** CALB^+^ cells (green). Scale bars, 100 μm. **g** Co-staining of S100β and TUJ1 shows presence of astrocytes in spinal organoids. Scale bars, 100 μm. **h** Quantitative-PCR analysis demonstrates a lack of dorsal cell types in the spinal organoids generated

The spinal cord is organized both rostro-caudally, as well as along the dorso-ventral axis. Motor neurons along the rostro-caudal axis are classified as cervical, brachial, thoracic, or lumbar depending on the muscle groups they innervate^8^. In order to determine if the spinal organoids recapitulate the diversity of neural cells along the rostro-caudal axis, we performed immunostaining for HOXB4 (cervical marker), HOXC8 (brachial/thoracic marker), and HOXC10 (lumbar marker) in the day 28 organoids. We found that while there were some clusters of HOXB4^+^ cells, many cells in the spinal organoid are HOXC8^+^ (Fig. 2a, b). HOXC10 staining was not detected, indicating that lumbar motor neurons are not present in the organoids (data not shown). Interestingly, our conventional 2D protocol resulted in a homogeneous layer of HOXB4-expressing cervical subtype cells (Supplementary Figure S2A), with no HOXC8 or HOXC10 immunoreactivity. The acquisition of caudal identity in the spinal cord is orchestrated by Growth and Differentiation Factor 11 (GDF11). Indeed, we measured increased expression of GDF11 in our organoid cultures over time while the 2D cultures were consistently lacking GDF11 expression (Supplementary Figure S2B). Quantitative PCR analysis also confirmed our immunostaining data, showing increased expression of HOXC8 over time in the organoids (Supplementary Figure S2C).

Along the dorso-ventral axis of the spinal cord, motor neurons are found in the ventral horns while sensory neurons are located in the dorsal horns. We confirmed that motor neurons in the spinal organoids were FOXP1 limb-innervating neurons (Fig. 2c) and cholinergic based on ISL1 and ChAT co-expression (Fig. 2d) at day 42. These neurons are also functional because co-culture of these organoids with mouse myotubes resulted in neurite outgrowth of about 650 μm within 3 days of co-culture (Supplementary Figures S2D, E) and myotube contractions could be observed (Supplementary Movie S1). C2C12 myotubes that were not co-cultured with organoids did not show any contraction. To confirm that neuromuscular junctions (NMJs) were formed, we labeled acetylcholine receptors at NMJs with alpha bungarotoxin (α-BTX) and observed close proximity of SMI-32^+^ axons with α-BTX stains by confocal microscopy (Supplementary Figure S2F).

Apart from motor neurons, we also detected the presence of other ventral spinal cord cells such as CHX10-expressing cells, indicating the formation of V2a interneurons (Fig. 2e; Supplementary Figure S1B). Presence of Calbindin^+^ cells, along with increased expression of PAX2 and LHX1 also suggests that V1 inhibitory interneurons known as Renshaw cells are present (Fig. 2f; Supplementary Figure S2C). Astrocytes, marked by S100β expression, can also be detected by day 35 (Fig. 2g). We however did not detect SOX10^+^ dorsal root ganglia progenitors or BRN3A^+^ sensory neurons in the organoids by immunostaining or qPCR, suggesting that our cultures are more representative of ventral spinal organoids (Fig. 2h). Additionally, we did not detect any increased expression of TLX3, a transcription factor expressed in the dorsal spinal cord (Fig. 2h), confirming the lack of dorsal cell types.

### SMA ventral spinal organoids do not show a defect in neurogenesis

**Fig. 3:**
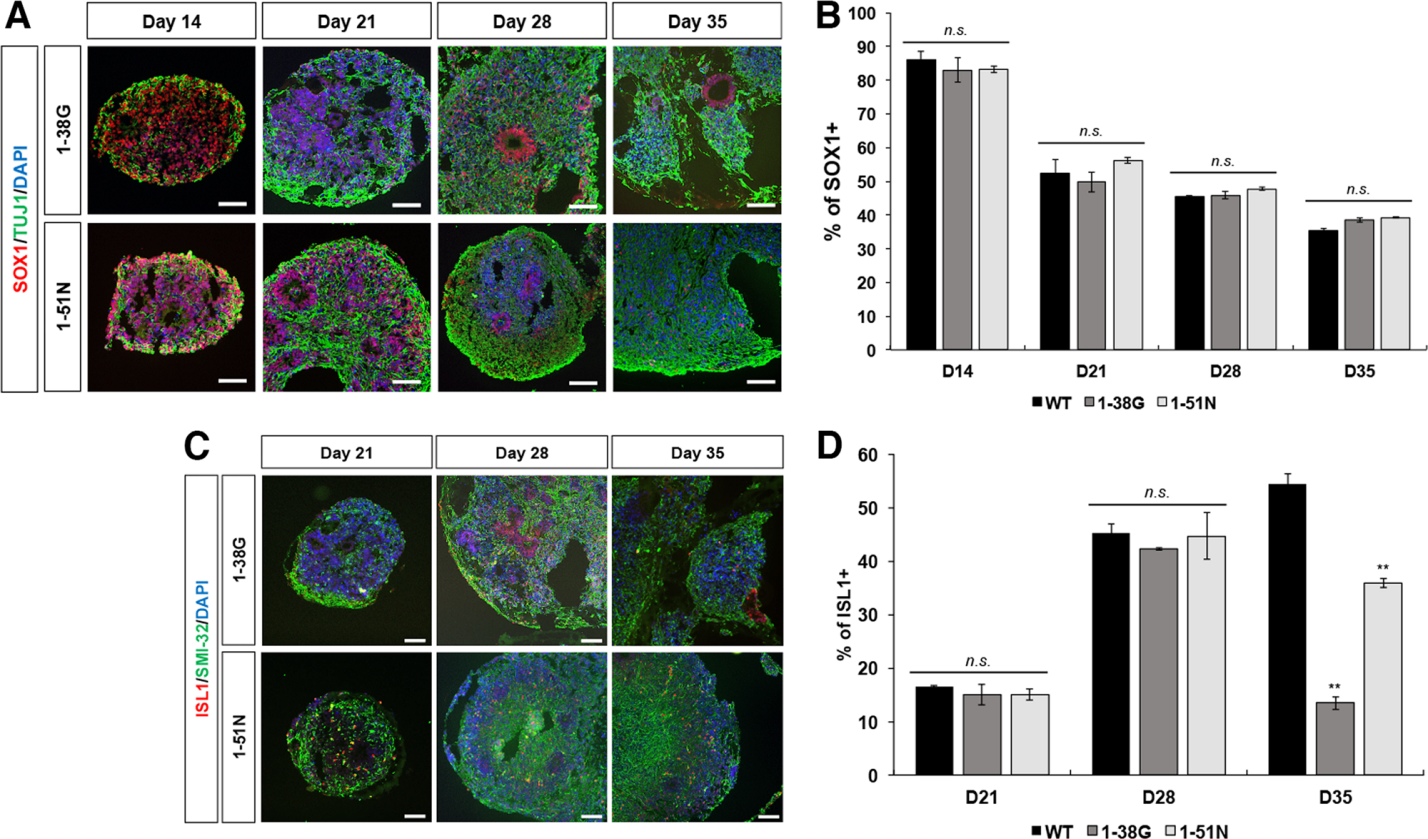
SMA organoids shows reduced motor neuron survival. **a** Co-staining of SOX1 (red) and TUJ1 (green) in SMA Type I (1-38 G) and SMA Type II (1-51 N) spinal organoids at respective time points. Cellular nuclei were counterstained with DAPI. Scale bars, 100 μm. **b** Quantification of SOX1^+^ levels percentage of SMA Type I and Type II spinal organoids at respective time points relative to total cell number. The values were not significant. **c** Representative images of SMA Type I and Type II spinal organoids at respective time points stained with ISL1 (red) and SMI-32 (green). Cellular nuclei were counterstained with DAPI. Scale bars, 100 μm. **d** Graph shows percentage of ISL1^+^ at day 21, 28, and 35 in SMA Type I and Type II spinal organoids relative to total cell number. **p < 0.01, n.s. non-significant

Using iPSCs derived from SMA Type I (1-38 G) and Type II (1-51 N) patients^9,10^, we derived ventral spinal organoids using the method described above. It has been suggested that SMA is also a neurodevelopmental disorder because histopathological analyses of spinal cords from patients have shown loss of anterior horn motor neurons, as well as immature and mismigrated neurons^11^. Therefore, to investigate plausible defects in neurogenesis in SMA organoids, we harvested organoids for cryosectioning and immunostaining every 7 days starting at day 14, until they reach day 35. Similarly, just like the wild type (WT) organoids (Fig. 1c), we saw the appearance of SOX1^+^ cells at day 14 that organizes into rosette structures by day 21 in SMA organoids (Fig. 3a, b). Reduced number of SOX1^+^ progenitor cells with rising appearance of ISL1^+^ motor neurons were also observed in the SMA organoids at day 21 (Fig. 3a, c). Correspondingly, TUJ1^+^ neurons appeared in day 14 and continued to be present in day 21, 28, and 35 SMA organoids (Fig. 3a). By day 28, 45.3% of all cells in WT organoids were made up of ISL1^+^ motor neurons. Likewise, we saw similar percentages of motor neurons in SMA organoids, with 42.2% in 1-38 G organoids and 44.7% in 1-51 N organoids (Fig. 3d). A cellular hallmark of SMA is the rapid degeneration of motor neurons^12,13^. When we cultured WT and SMA organoids to day 35, we observed that while the WT motor neurons increased slightly to 54.5% in the organoid, motor neuron numbers declined to 13.5% in 1-38 G organoids (*p* = 0.0015) and 35.9% in 1-51 N organoids (*p* = 0.0061) (Fig. 3c, d). This indicates that SMA motor neurons were unviable shortly after formation, with the Type I organoids showing a more severe degenerative phenotype compared to the Type II organoids. This is similar to our observations for motor neurons derived using a conventional differentiation protocol^1^, and confirms that ventral spinal organoids recapitulate the cellular features of SMA.

### SMA motor neurons express high levels of cell cycle CDKs and cyclins

**Fig. 4:**
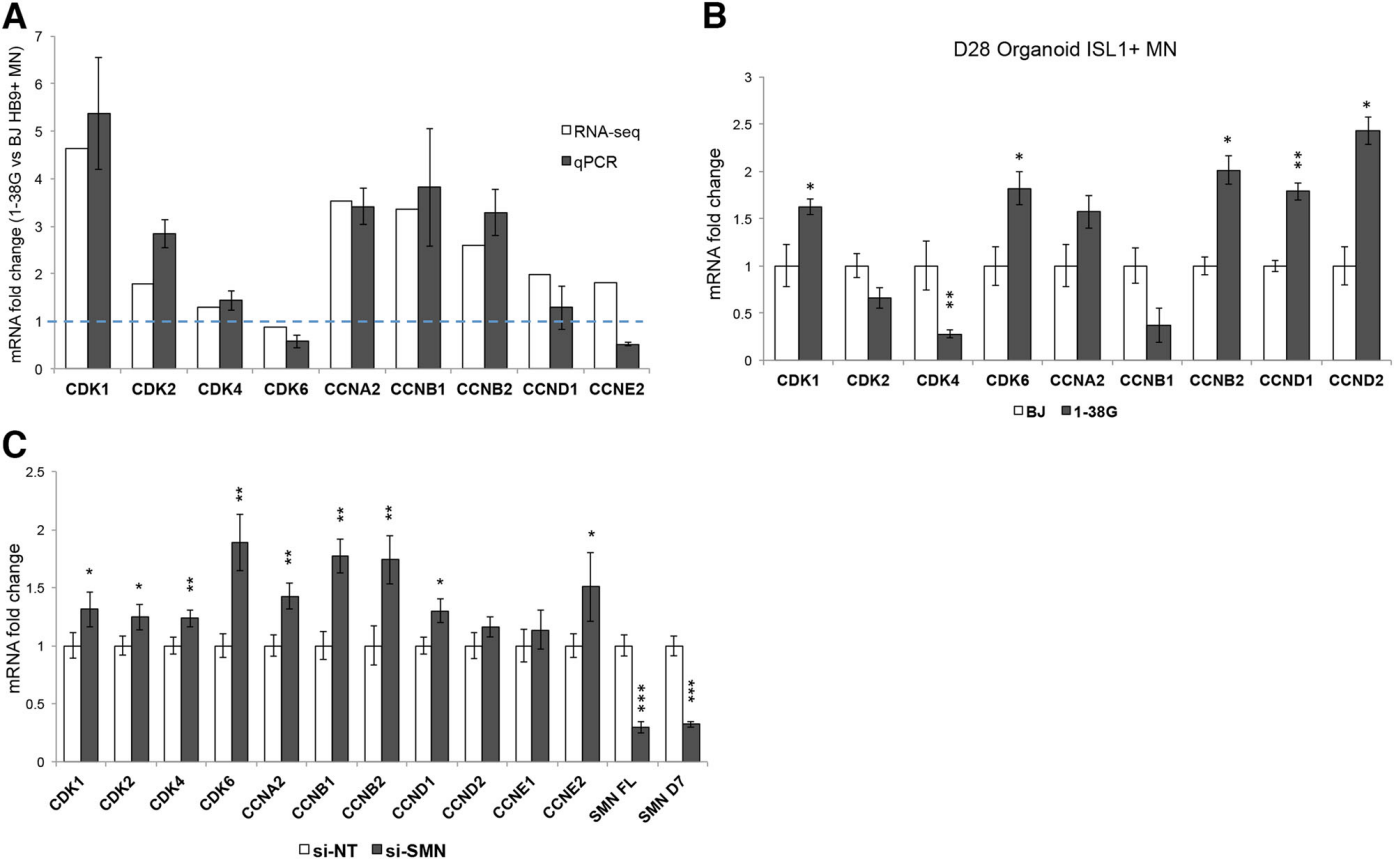
Cell cycle genes are upregulated in SMA motor neurons. **a** Motor neurons were purified based on HB9 immunoreactivity. mRNA expression levels of CDKs and cyclins measured by RNA-seq and qPCR respectively. Graph shows fold change comparing SMA HB9^+^ motor neurons to wild-type HB9^+^ motor neurons. The blue dotted line indicates relative expression of wild-type HB9^+^ motor neurons. **b** qPCR analysis of ISL1^+^ motor neurons derived from day 28 organoids. Graph shows mRNA fold change, relative to BJ ISL1^+^ motor neurons. **c** Knockdown of SMN in wild-type motor neuron cultures revealed upregulation of cell cycle genes. *p < 0.05; **p < 0.01, and ***p < 0.001

In a previous study, we profiled the expression of purified HB9^+^ motor neurons derived from WT and 1-38 G iPSCs and found specific transcriptional events in diseased motor neurons^1^. The ability to isolate pure populations of motor neurons for RNA-seq circumvented the problem of intrinsic heterogeneity of iPSC-derived cultures, which was detrimental to whole transcriptome analyses because specific yet small changes in the diseased motor neuron population are often masked by the other contaminating cell types in the culture. From the RNA-seq study, we found that mRNAs corresponding to CDK1, CDK2, CDK4, Cyclins A2, B1, B2, and D1 were upregulated in purified 1-38 G motor neurons compared to controls. To confirm this, we differentiated SMA and wild-type iPSCs towards the spinal motor neuron fate^1^, and performed quantitative PCR on cDNA samples of purified HB9^+^ motor neurons by FACS sorting. This sorting removed variability of differentiation efficiencies between different cell lines and revealed higher expression of the cell cycle genes *CDK1*, *CDK2*, *CCNA2*, *CCNB1*, and *CCNB2* (Fig. 4a), confirming our RNA-seq results. We also isolated RNA from purified ISL1^+^ motor neurons in organoids made from 1-38 G and BJ iPSCs at day 28 for qPCR analysis and found similar upregulation in several CDKs and cyclins (Fig. 4b). This confirms that the spinal organoids recapitulated the molecular phenomenon that was observed in our previous 2-dimensional cultures.

We postulate that loss of SMN protein was responsible for the upregulation of cell cycle genes, and to investigate this, we performed siRNA-mediated knockdown of SMN in WT motor neuron cultures. Quantitative PCR analysis revealed that loss of SMN was indeed responsible for the upregulation of Cyclins A2, B1, and B2 as we observed in the SMA iPSC-derived motor neurons. In addition, depletion of SMN also resulted in upregulation of CDK6 and Cyclin E2 expression in the motor neuron cultures (Fig. 4c).

### Low levels of SMN trigger cell cycle re-entry in motor neurons

**Fig. 5:**
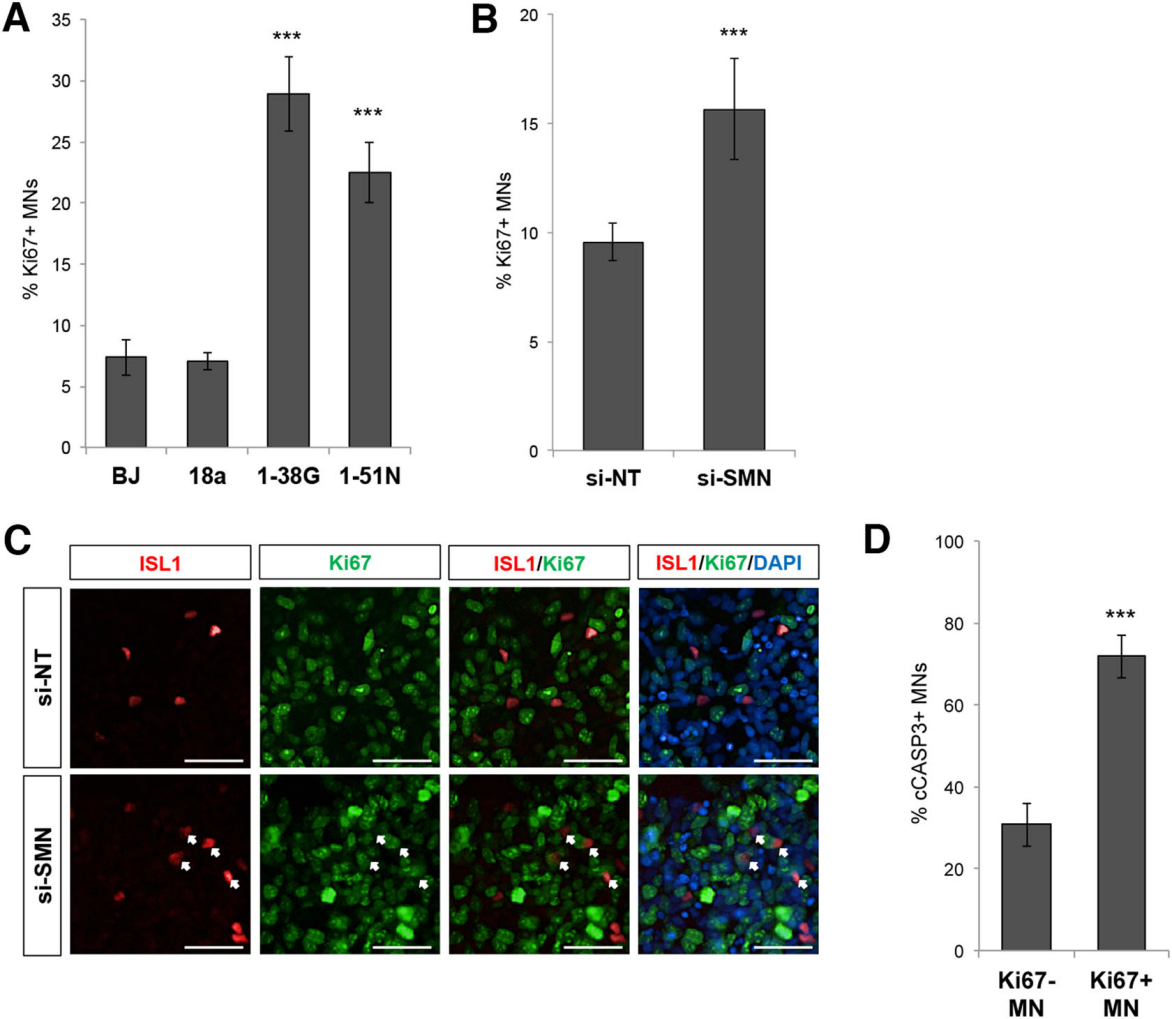
Loss of SMN reactivates the cell cycle. **a** Ki67 and ISL1 immunostaining analysis of wild-type (BJ iPS and 18a), SMA Type I (1-38 G), and SMA Type II (1-51 N) motor neuron cultures at day 28. The percentages of ISL1^+^Ki67^+^ cells amongst all ISL1^+^ motor neurons are shown. **b** Knockdown of SMN in wild-type cell line (BJ-iPS) increased the percentage of ISL1^+^ motor neurons co-expressing Ki67. **c** Co-staining of ISL1 (red) and Ki67 (green) showing increased Ki67^+^ cells upon SMN knockdown in BJ-iPS motor neuron cultures. Cellular nuclei were counterstained with DAPI. Scale bars, 100 μm. **d** Ki67 and cCASP3 immunostaining analysis of wild-type motor neurons demonstrated higher cCASP3 expression in Ki67^+^ motor neurons than Ki67^−^ motor neurons. ***p < 0.001

Depletion of SMN resulting in cell cycle gene activation suggests that motor neurons deficient in SMN aberrantly re-enter the cell cycle. To confirm this, we investigated the cell cycle status of motor neurons in WT and SMA cultures by determining the percentage of motor neurons that are co-expressing the proliferative marker Ki67. While 7−8% of wild-type ISL1^+^ motor neurons co-label Ki67, we found that 28.9 and 22.5% (*p* < 0.01) of SMA Type I and Type II motor neurons co-express Ki67, respectively (Fig. 5a). Since motor neurons are postmitotic cells and Ki67 expression is a hallmark of dividing cells, this indicates that loss of SMN triggers cell cycle re-entry in motor neurons. Furthermore, depletion of SMN protein by siRNAs in wild-type BJ-iPS motor neuron cultures also led to a significant increase in the percentage of Ki67-expressing motor neurons (Fig. 5b, c).

To demonstrate that reactivation of the cell cycle is deleterious to motor neurons, we compared the rate of apoptosis of Ki67^+^ motor neurons versus Ki67^−^ motor neurons by co-staining with cleaved Caspase-3 (cCASP3). While 30.7% of Ki67^−^ motor neurons co-express the apoptotic marker cCASP3, we found that 71.8% of Ki67^+^ motor neurons co-express cCASP3, indicating that reactivation of the cell cycle in motor neurons resulted in apoptosis (Fig. 5d).

### Inhibition of CDKs rescue SMA motor neurons

Since SMN-deficient motor neurons re-activate the cell cycle, we wondered if inhibition of cell cycle components such as CDKs would rescue SMA motor neurons from cell death. We first treated SMA motor neurons for 3 days with a pan-CDK inhibitor (CDKi) known to selectively inhibit CDK1/Cyclin B, CDK2/Cyclin E, and CDK4/Cyclin D1 complexes, and found that it was able to promote SMA motor neuron survival by up to 30% compared to a DMSO control (Supplementary Figure S3).

**Fig. 6:**
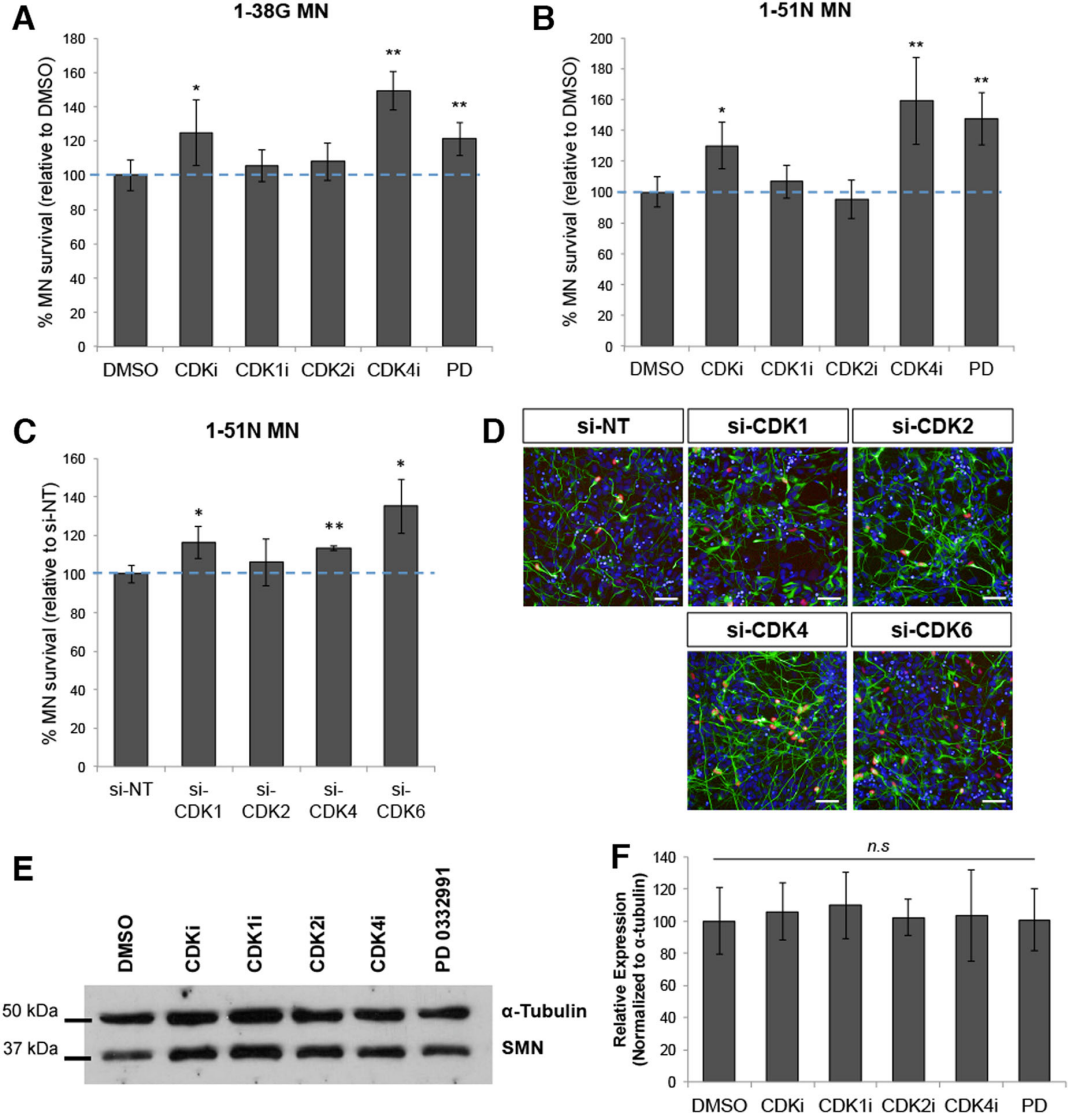
Inhibition of CDKs prolongs SMA motor neuron survival. **a** Graphical representation of ISL1^+^ SMA type I motor neurons (1-38 G) treated with various CDKs inhibitors treatment. The blue dotted line indicates percentage of motor neurons relative to DMSO-treated motor neurons. **b** Quantification of ISL1^+^ SMA type II motor neurons (1-51 N) treated with various CDKs inhibitors treatment. The blue dotted line indicates percentage of motor neurons relative to DMSO-treated motor neurons. **c** ISL1 immunostaining analysis of various CDKs knockdown in SMA type II motor neurons. The blue dotted line indicates percentage of motor neurons survival relative to non-targeting siRNA treated motor neurons. **d** Representative images of SMA type II motor neurons treated with various CDKs siRNA and stained with ISL1 (red) and SMI-32 (green). Cellular nuclei were counterstained with DAPI. Scale bars, 50 μm. **e** Western blot of SMA type II motor neurons treated with various CDKs inhibitors, indicating that SMN levels remained the same. **f** Quantification of SMN levels of SMA type II motor neurons treated with various CDKs inhibitors relative to α-tubulin expression. The values were not significant. *p < 0.05 and **p < 0.01

To expand our study on CDK inhibition and its effects on neuroprotection in SMA, we carried out a series of motor neuron survival experiments using specific inhibitors of CDKs: CDK1 Inhibitor (CDK1i), CDK2 Inhibitor II (CDK2i), CDK4 Inhibitor (CDK4i), as well as PD 0332991 (PD), a CDK4/6 inhibitor. We found that while CDK1 and CDK2 inhibition had no significant effect on SMA motor neuron survival compared to DMSO, inhibition of CDK4 and CDK6 by CDK4i and PD resulted in up to 50% increase in motor neuron survival (Fig. 6a, b) in both the SMA Type I and II cultures.

In addition to the small molecule inhibitor study, we performed siRNA-mediated knockdown of each specific CDK on our SMA motor neurons (Supplementary Figure S4) and quantified motor neuron numbers following siRNA treatment. Confirming results from the CDK small molecule inhibitor experiment, we found that specific knockdown of *CDK4* and *CDK6* led to 27.1% and 18.4% increase in SMA motor neuron survival, respectively, while depletion of *CDK1* and *CDK2* yielded insignificant results (Fig. 6c, d). Importantly, this neuroprotection was not due to an elevated SMN protein expression that could have resulted from CDK inhibition or knockdown (Fig. 6e, f).

### Motor neuron death in SMA ventral spinal organoids reversed with CDK inhibitor

**Fig. 7:**
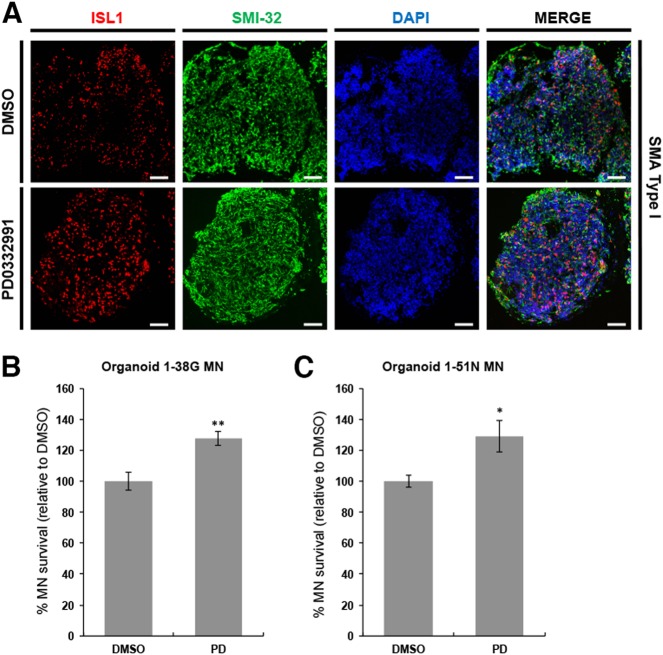
CDK inhibitor reversed motor neuron death in SMA spinal organoids. **a** Co-staining of ISL1 (red) and SMI-32 (green) in SMA type I spinal organoids treated with DMSO and PD0332991. Cellular nuclei were counterstained with DAPI. Scale bars, 100 μm. **b** SMA type I and **c** SMA type II spinal organoids shows increased MN survival. *p < 0.05 and **p < 0.01

Since neural organoids represent a more “in vivo-like” culture system compared to conventional two-dimensional cultures, we investigate if ventral spinal organoids could be a good model for testing of therapeutic compounds. As a proof of principle, we tested the efficacy of PD 0332991 in reversing the SMA motor neuron degenerative phenotype. To this end, we treated SMA organoids at day 28 with either DMSO (control) or 0.1 μM PD for 7 days, and performed histology to assess the treated and control organoids at day 35. We analyzed at least five organoids in each treatment condition by cryosectioning and immunostaining and found that SMA ventral spinal organoids remained highly neuronal based on SMI-32 labeling. We also found that ISL1^+^ motor neuron survival in PD-treated SMA organoids was increased by 27.6% in 1-38 G and 29.1% in 1-51 N compared to DMSO-treated organoids (Fig. 7a–c). This confirms that PD 0332991 was specific in rescuing SMA motor neurons, even in the context of a ventral spinal organoid culture.

## Discussion

In this study, we report the formation of ventral spinal organoids from iPSCs derived from SMA and healthy individuals to study neurodevelopmental, as well as neurodegenerative aspects of the disease. Detailed characterization of these organoids revealed that motor neurons, astrocytes, as well as interneurons that form part of the motor circuit were derived, mimicking the microenvironment in vivo. This would be especially useful for studying hyperexcitability of motor neurons in diseases such as SMA and Amyotrophic Lateral Sclerosis (ALS)^14,15^. It has been demonstrated that this abnormal neuronal firing can be caused by changes in the premotor circuits, where interneurons such as Renshaw cells exert inhibitory feedback control on motor neuron firing^16,17^. However, dorsal cell types such as sensory neurons and dorsal interneurons were absent, limiting the use of this ventral spinal organoids to the studying of motor neurons and premotor circuits rather than sensory-motor connectivity.

Organoids have immense potential for studying human neural development, and various neural organoids, including cerebral (forebrain) organoids, retinal organoids, and midbrain organoids^18–20^ have been generated for that purpose. Therefore, we made use of our spinal organoids to first address neurodevelopmental processes in SMA. It remains controversial whether SMA is a neurodevelopmental disease. In the most severe forms of SMA, the very early onset of disease before 6 months of age and the lack of developmental milestones achievement suggest a developmental defect of the motor unit^21,22^. Presence of fetal forms of acetylcholine receptors in NMJs of SMA Type I patients, as well as loss of spinal motor neurons and presence of misguided and immature motor neurons appear to support that^23–25^. It has also been reported that SMA mice have fewer spinal motor neurons at birth compared to healthy littermates^26^, further suggestive of a neurogenesis defect. Therefore, using spinal organoids, we sought to investigate manifestations of neurodevelopmental phenotypes in SMA.

Time-coursed analysis of healthy and SMA ventral spinal organoids revealed similar neurogenesis dynamics, where similar percentages of healthy and SMA SOX1^+^ neural progenitor cells were observed at each time point, indicative of a normal neural progenitor population. Likewise, similar percentages of healthy and SMA ISL1^+^ motor neurons were measured at days 21 and 28, highlighting that motor neuron formation is not impaired. Interestingly, SMA motor neurons rapidly degenerate between days 28 to 35, and the severity of motor neuron loss correlated with their clinical classifications, demonstrating that these spinal organoids have the capacity to model the spectrum of motor neuron degenerative phenotype with cells derived from Type I and Type II SMA patients.

While neural organoids have been widely used to model neurodevelopmental disorders, their utility in studying neurodegenerative disorders is less common. As effective models of neurodegeneration, we further tested the utility of spinal organoids as disease models by conducting two-dimensional (2D) and organoid experiments simultaneously. Using SMA patient-derived motor neurons and SMN knockdown approaches, we found that SMN-deficient motor neurons aberrantly reactivate the cell cycle, in agreement with recently published reports that SMN deficiency in motor neurons induces p53 activation^27^. It has been previously reported that postmitotic neurons re-enter cell cycle as a response to DNA damage and a simultaneous increase in p53 expression^28^. Importantly, we also found that blocking cell cycle progression by means of a CDK4/6 inhibitor significantly prolonged SMA motor neuron survival both in 2D and ventral spinal organoid cultures. This confirmed that neural organoids are amenable for small molecule screening approaches and could be considered as an additional screening step before moving into in vivo models.

## Experimental procedures

### Culture and differentiation of human pluripotent stem cells

Wild-type BJ fibroblast-derived iPSCs (BJ-iPS) and SMA patient-derived iPSCs (Type II SMA 1-51 N and Type I SMA 1-38 G) were cultured feeder-free on Matrigel-coated plates in MACS iPS-Brew media (Miltenyi Biotec). Routine passaging using ReLeSR (Stem Cell Technologies) is performed once every 6-7 days. Pluripotent stem cells were differentiated towards the spinal motor neuron fate following established protocols described previously^1^. Spinal organoids were made by dissociating iPS cells into single cells, and seeded either 10,000 or 30,000 cells per well in a 96-well low-attachment plate. Eventually we used 30,000 cells per well because that resulted in better derivation of mature spinal cell types (Supplementary Figure S1). The embryoid bodies were then encapsulated in 15 μl Matrigel droplets at day 10 before transferring to spinner flasks at day 14 for neuronal maturation in the presence of growth factors BDNF and GDNF. Organoids can be maintained for at least 90 days, although in this manuscript, organoids were harvested by day 42 for analysis (Supplementary Figure S1).

### RNA extraction and expression analysis

Cells were harvested in Trizol reagent for RNA extraction following manufacturer’s instructions. Purified RNA was converted to cDNA using the High-Capacity cDNA Reverse Transcription kit (Ambion), and quantitative PCR (qPCR) was performed on the QuantStudio 5 Real-Time PCR System using FAST SYBR Master mix (all from Applied Biosystems). Gene expressions were normalized to GAPDH and ACTB expression unless otherwise stated. A list of the qPCR primers used is provided in Supplementary Table S1.

### RNA interference in motor neuron cultures

Motor neuron cultures were dissociated with Accutase and seeded at 75,000 cells per well in a 96-well plate. Non-targeting siRNA or siRNAs against genes of interest were individually complexed with Lipofectamine RNAiMAX (Invitrogen) following manufacturer’s instructions. For each well, 10 pmol of siRNAs and 0.5 μl of Lipofectamine RNAiMAX were used. Cells were either harvested for RNA and protein analyses or fixed for immunostaining three days after siRNA transfection.

### Treatment of motor neuron cultures with small molecule inhibitors

CDKi hydrochloride (Sigma), specific CDK1 inhibitor (Santa-Cruz), specific CDK2 inhibitor (Santa-Cruz), specific CDK4 inhibitor (Santa-Cruz), and PD 0332991 (Santa-Cruz), were reconstituted in DMSO and diluted in media at the desired concentrations: CDKi (10 μM), CDK1i (0.1 μM), CDK2i (1 μM), CDK4i (0.1 μM) PD (0.1 μM). Motor neurons at day 23 were plated at 75,000 cells per well of a 96-well plate. Treatment with the respective small molecules began at day 25, for a total of 3 days. Biological triplicates were performed with a minimum of five technical replicates each.

### Treatment of SMA spinal organoids with small molecule inhibitors

SMA spinal organoids were treated with either DMSO or PD 0332991 (0.1 uM) on a low-attachment plate at day 28 for a total of 7 days. SMA spinal organoids were then harvest at Day 35 for cryosectioning.

### SDS-PAGE and western blot

Protein lysates were resolved in 12% SDS-PAGE gels in Tris-Glycine-SDS buffer. Proteins were then transferred to a PVDF membrane and blocked in buffer containing 5% milk. Primary antibodies were diluted in 5% milk and incubated with the membranes overnight at 4 °C. The following primary antibodies (and their respective dilutions) were used: mouse SMN (1:1000) (BD Pharmingen, 610647), mouse α-tubulin (1:500) (Santa Cruz Biotechnologies, sc-32293). Membranes were washed thrice in TBST buffer. The corresponding horseradish peroxidase secondary antibodies (Santa Cruz) were then diluted 1:2000 in 5% milk and incubated at room temperature for 90 min. Blots were washed thrice before exposing to ECL for imaging.

### Immunofluorescence, image acquisition, and image analysis

Cells were fixed in 4% paraformaldehyde for 15 min, permeabilized in 0.1% Triton X-100 for 15 min, and blocked in buffer containing 5% FBS and 1% BSA for an hour at room temperature. Primary antibodies were diluted in blocking buffer and incubated overnight at 4 °C. The following primary antibodies (and their respective dilutions) were used: rabbit SOX1 (1:1000) (Abcam, ab87775), mouse Nestin (1:1000) (Abcam, ab22035), rabbit ISL1 (1:1500) (Abcam, ab109517), rabbit cleaved Caspase-3 (1:1000) (Cell Signaling Technology, #9661), mouse Ki67 (1:1500) (Cell Signaling Technology, #9449), mouse SMI-32 (1:1000) (Calbiochem, NE-1023), mouse SMN (1:400) (BD Pharmingen, 610647), rabbit Ki67 (1:250) (Abcam, ab16667), mouse TUJ1 (1:2000) (Biolegend, #801202), goat SOX10 (1:100) (Santa Cruz Biotechnologies, sc-17342), rabbit HOXB4 (1:200) (Abcam, ab133521), rabbit HOXC8 (1:200) (Abcam, ab86236), rabbit Calbindin (1:1000) (Abcam, ab11426), mouse FoxP1 (1:100) (R&D Systems, MAB45341), and sheep Chx10 (1:200) (Abcam, ab16141). The cells were washed thrice in PBS. The respective secondary antibodies (Molecular Probes, Invitrogen) were diluted 1:1500 in blocking buffer and incubated at room temperature, in the dark, for 90 min. DAPI was used at 0.1 μg/ml to visualize cellular nuclei.

Images of cultured cells on 96-well plates were acquired using the high content microscope Operetta (Perkin Elmer) using the ×20 objective. Image analyses including cell counts and intensity measurements were performed using Columbus (Perkin Elmer). Nuclei were detected based on DAPI staining, with dead cells filtered based on abnormally high DAPI intensity and small (<20 μm^2^) nuclei area. Intensity of ISL1 staining within nuclei was determined and a cut-off above background intensity was used to identify motor neurons.

Spinal organoids were fixed in 4% PFA overnight, and dehydrated in 15% sucrose and 30% sucrose for 24 h each before cryosectioning at 10 μm per section. These were then stained respectively with antibodies listed above and images were acquired with an upright fluorescence microscope (Nikon Eclipse Ni) using the ×10 objective. ISL1^+^ and SOX1^+^ cells were quantified by automated counting performed by image analysis software (ImageJ, NIH). All quantifications were normalized to total DAPI counts.

### Statistical analyses

At least three biological replicates were performed for each experiment. Statistical analysis comparing two groups were performed by means of a two-tailed unpaired Student’s *t* test. *P* values lower than 0.05 were considered significant. All results are presented as mean ± standard deviation unless otherwise specified.

## Electronic supplementary material

Supplemental Data

Supplemental Data
