# Supplementary material for: Cell cycle inhibitors protect motor neurons in an organoid model of Spinal Muscular Atrophy
Source: Cell Death Dis. 2018 Oct 27;9(11):1100. doi: 10.1038/s41419-018-1081-0 (PMC6204135; doi:10.1038/s41419-018-1081-0)
Supplement: Supplementary file 1 — Supplemental Data [file 41419_2018_1081_MOESM1_ESM.docx]

**SUPPLEMENTARY INFORMATION**

**Cell cycle inhibitors protect motor neurons in an organoid model of Spinal Muscular Atrophy**

Jin Hui Hor^1,2^, Eunice Shi-Yi Soh^1,3^, Li Yi Tan^1^, Valerie Jing Wen Lim^1^, Munirah Mohamad Santosa^1^, Winanto^1,3^, Beatrice Xuan Ho^1,2^, Yong Fan^6^, Boon-Seng Soh^1,2,6#^, Shi-Yan Ng^1,4,5,6#^

^1^ Institute of Molecular and Cell Biology, 61 Biopolis Drive, Singapore 138673

^2^ Department of Biological Sciences, National University of Singapore, 14 Science Drive 4, Singapore 117543

^3^ School of Biological Science, Nanyang Technological University, Singapore 637551

^4^ National Neuroscience Institute, 11 Jalan Tan Tock Seng, Singapore 308433

^5^ Department of Physiology, National University of Singapore, 28 Medical Drive, Singapore 117456

^6^ The Third Affiliated Hospital of Guangzhou Medical University, Guangzhou, 510150, China

# Correspondence to Shi-Yan Ng ([syng@imcb.a-star.edu.sg](mailto:syng@imcb.a-star.edu.sg)) and Boon-Seng Soh ([bssoh@imcb.a-star.edu.sg](mailto:bssoh@imcb.a-star.edu.sg))

**Supplementary Methods**

**Co-culture of spinal organoids with mouse myotubes**

The C2C12 cell line (ATCC) was maintained in myoblast media consisting of DMEM (high glucose) with 20% fetal bovine serum (FBS) on gelatin-coated dishes. To differentiate these towards myotubes, confluent C2C12 myoblasts were cultured in DMEM (high glucose) with 2% FBS on Matrigel-coated plates. 7 days after differentiation, myotubes were visibly formed, and were conditioned in N2B27 media a day before seeding organoids. 5 organoids at day 21 were seeded into each well of a 6-well plate and extensive neurite elongation was observed 3 days after co-culture. A video was taken at day 3 of co-culture (**Supplementary Movie S1**) where myotube contraction was observed in multiple parts of the well adjacent to the organoids. Neurite outgrowth was measured by image analysis software (ImageJ, NIH).

**Visualization of neuromuscular junctions**

At day 28, co-cultures were washed twice with PBS and alpha-Bungarotoxin (BTX) labeling (Life Technologies, B35451) of AChRs was performed on the co-cultures with 5µg/ml in PBS for 15 mins at RT. After BTX staining, cells were washed twice with PBS prior to acetone/methanol (ratio 1:1) fixation for 6 mins at −20°C. Permeabilization was done in 0.1% Triton X-100 for 15 minutes and blocked in buffer containing 5% FBS and 1% BSA for an hour at room temperature. Primary antibodies were diluted in blocking buffer and incubated overnight at 4 °C. The following primary antibodies (and their respective dilutions) were used: mouse SMI-32 (1:1000) (Calbiochem, NE-1023). Cells were washed thrice in PBS. The respective secondary antibodies (Molecular Probes, Invitrogen) were diluted 1:1500 in blocking buffer and incubated at room temperature, in the dark, for 90 minutes. DAPI was used at 0.1 μg/ml to visualize cellular nuclei. Images of neuromuscular junctions from the co-cultures were acquired with an inverted confocal microscope (Olympus FLUOVIEW FV1000) using the 20x objective.

**Supplementary Figures**


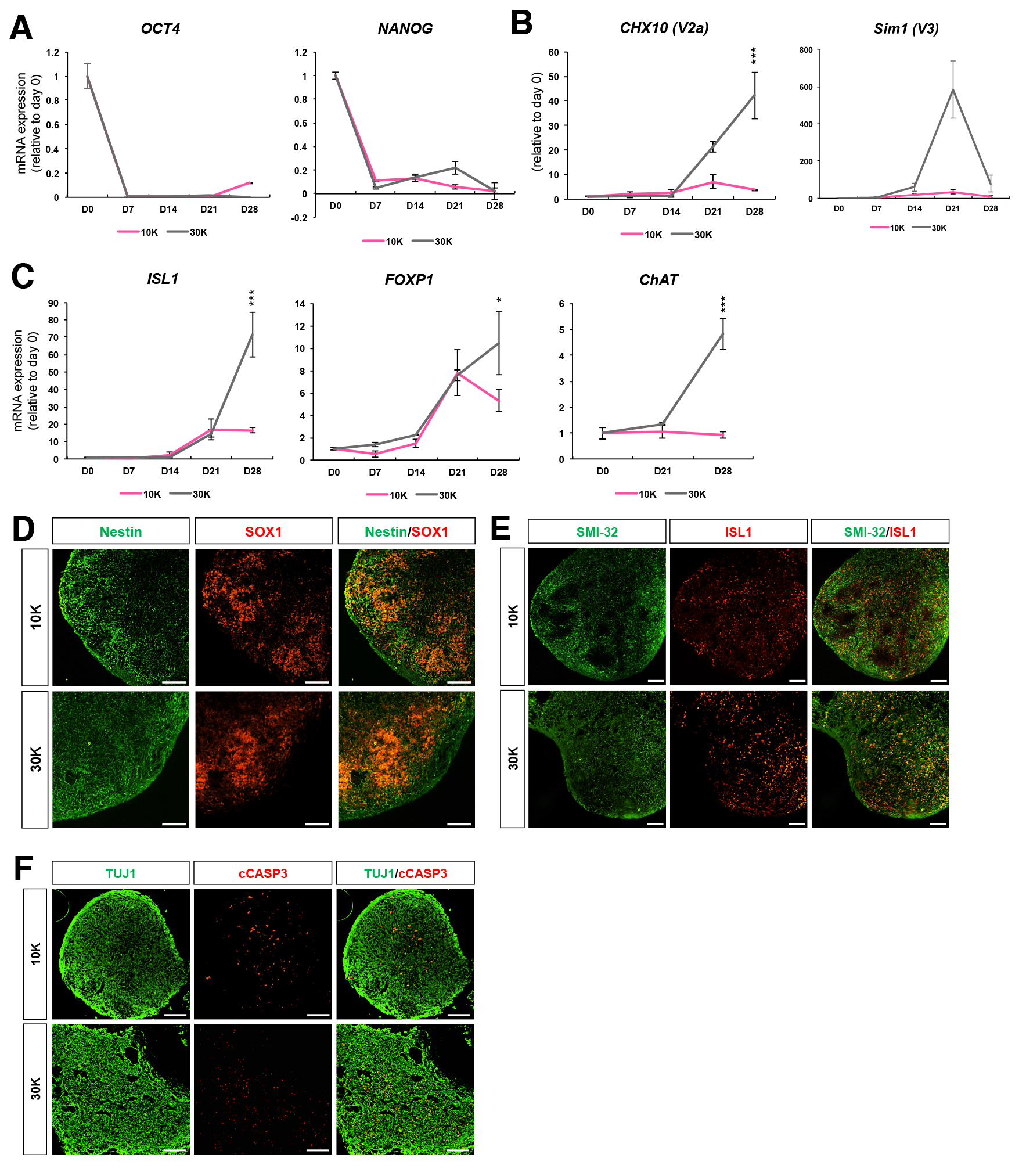


**Supplementary Figure S1: Comparison of spinal organoids made with 10,000 versus 30,000 cells revealed that higher cell density promotes motor neuron maturation and cellular diversity.** (A) qPCR plots showing similar reduction in pluripotency markers in organoids made with 10,000 and 30,000 cells. (B) Significantly higher expression of V2a marker CHX10 and V3 marker Sim1 were observed at days 21 and 28 in the 30,000-cell condition. (C) Motor neuron markers ISL1, FOXP1 and ChAT were also significantly increased in 30,000-cell conditions. (D-F) Immunostaining of organoids at day 28 suggests normal neural progenitor and motor neuron populations in 10,000- and 30,000-cell conditions. Staining with cleaved Caspase-3 (cCASP3) demonstrated that the core of the organoids were also not apoptotic.


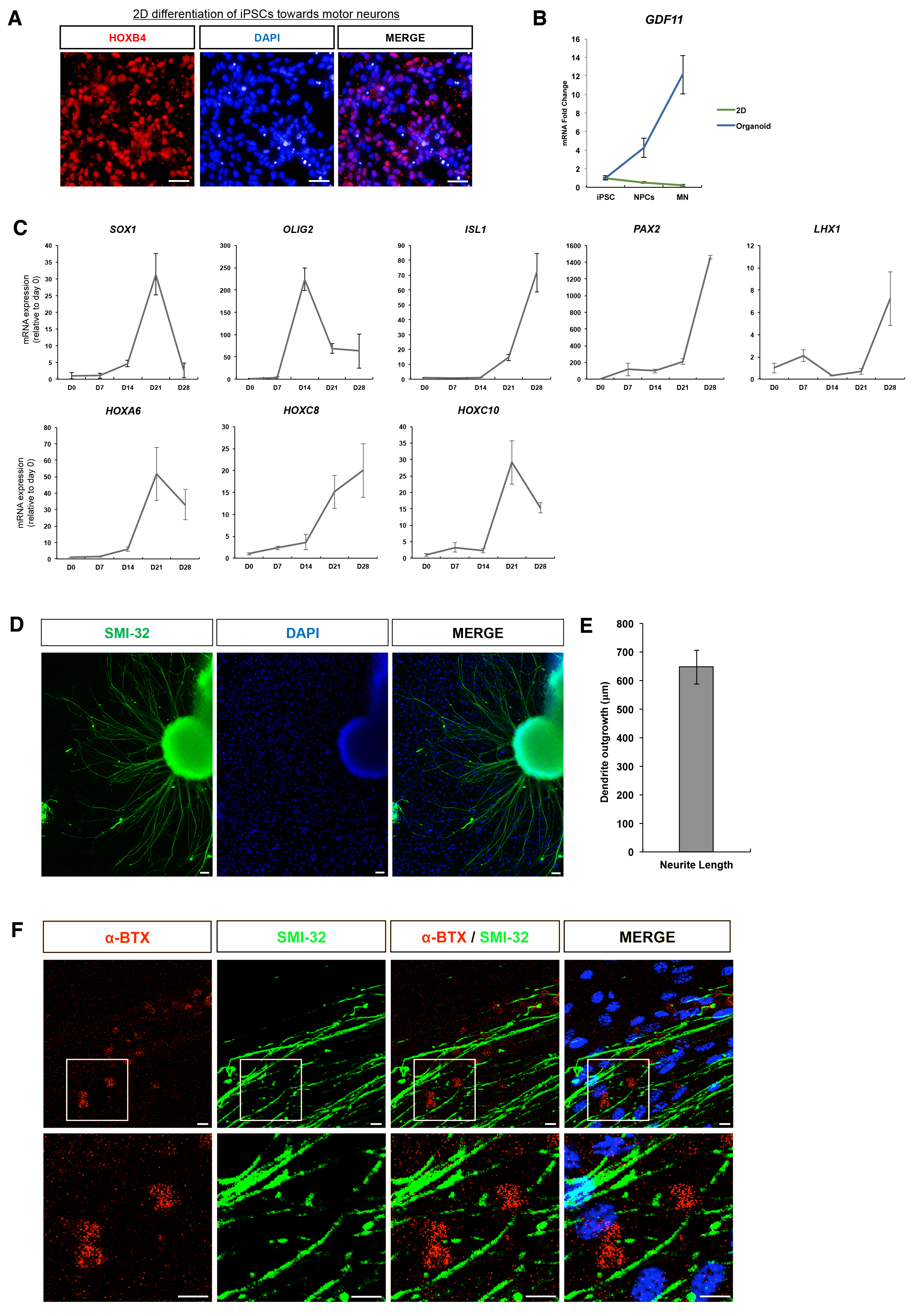


**Supplementary Figure S2: Molecular and functional characterization of ventral spinal organoids.** (A) Immunostaining image showing homogeneous generation of HOXB4+ cervical spinal cell types. (B) Time-coursed qPCR analysis showing increased GDF11 expression in organoid cultures, but not in conventional 2D cultures. (C) Time-coursed qPCR analysis of ventral spinal organoids revealed diversity of cell types within the organoids, including derivation of thoracic (HOXA6+), brachial (HOXC8+) and slight increase in lumbar (HOXC10) expression. (D) Motor neurons (stained with SMI-32) were co-cultured on mouse myotubes differentiated from C2C12 cell line. (E) Mean neurite lengths from the SMI-32 motor neurons in (D) were determined using Neurite Tracer in ImageJ. (F) Visualization of NMJs by staining acetylcholine receptors with α-BTX and co-staining adjacent motor axons with SMI32.


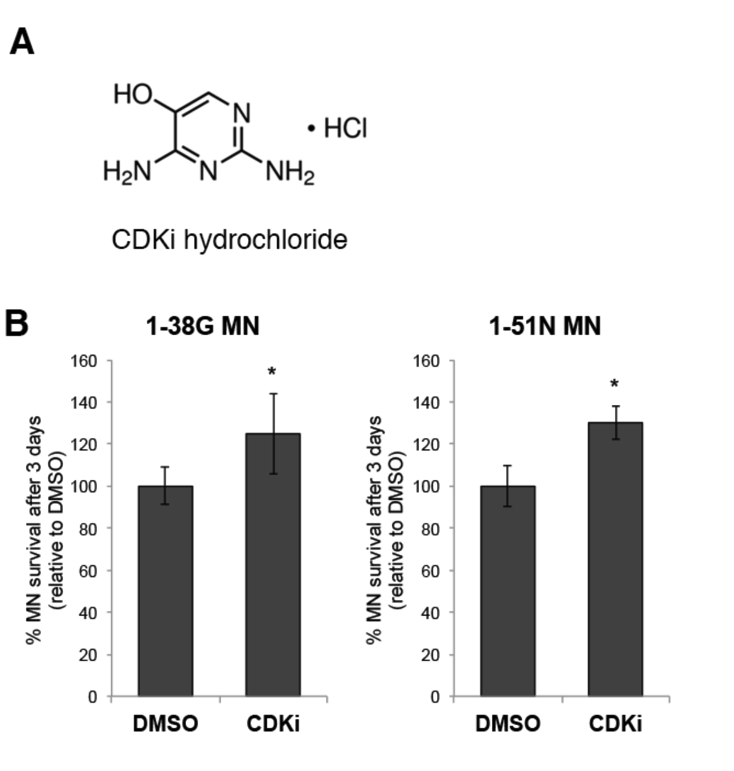


**Supplementary Figure S3: Chemical inhibition of CDKs prolongs SMA motor neuron survival.** (A) Chemical structure of pan-CDK inhibitor. (B) ISL1 immunostaining analysis demonstrated increased percentage of ISL1^+^ SMA type I and type II motor neurons (1-38G and 1-51N respectively) upon pan-CDK inhibitor treatment for 3 days. * indicates p < 0.05.

**
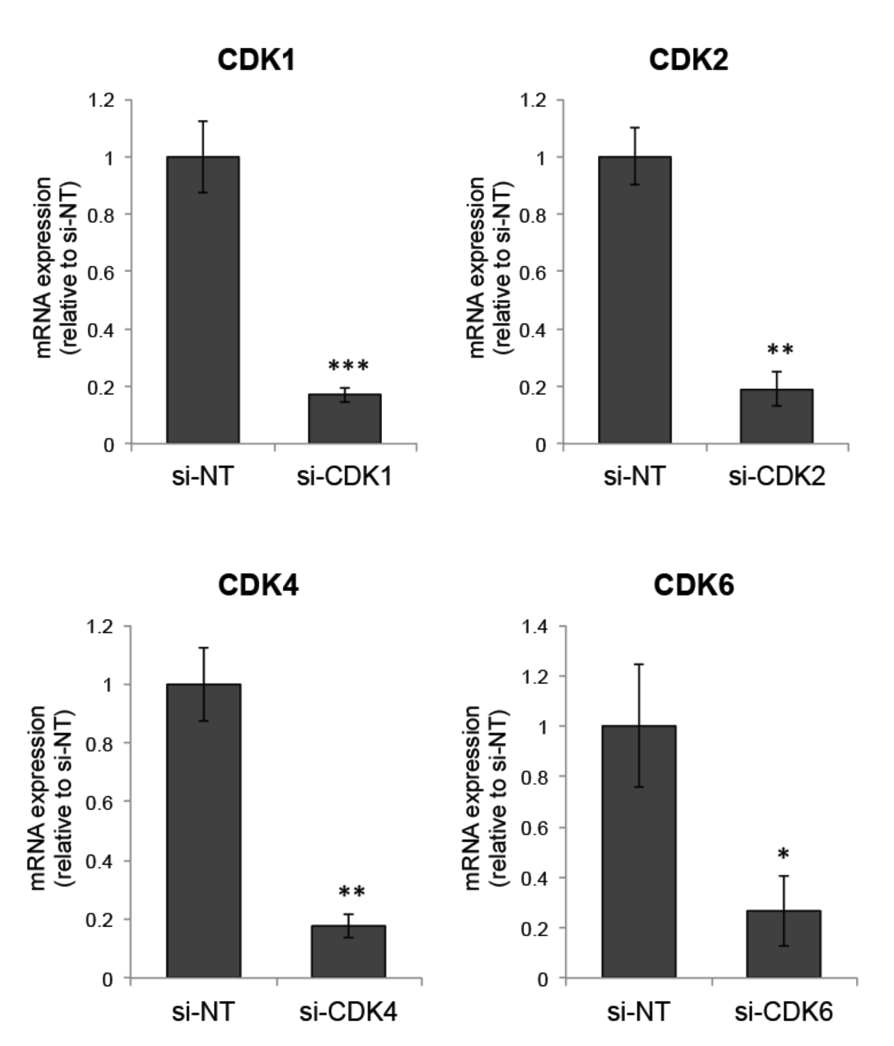
**

**Supplementary Figure S4: Efficient siRNA-mediated knockdown of CDKs in human motor neuron cultures.** qPCR analyses confiming efficient knockdown of CDK1, CDK2, CDK4 and CDK6 respectively, compared to a non-targeting siRNA control, in SMA motor neurons. * indicates p < 0.05; ** indicates p < 0.01 and *** indicates p < 0.001.

**Supplementary Tables**

**Supplementary Table S1**: List of human primers used in qPCR studies

| **Target gene** |  | **Primer sequence (5' to 3')** |
| --- | --- | --- |
| **CDK1** | F: | TGG ATC TGA AGA AAT ACT TGG ATT CTA |
|  | R: | CAA TCC CCT GTA GGA TTT GG |
| **CDK2** | F: | AAA GCC AGA AAC AAG TTG ACG |
|  | R: | GTA CTG GGC ACA CCC TCA GT |
| **CDK4** | F: | GTG CAG TCG GTG GTA CCT G |
|  | R: | TTC GCT TGT GTG GGT TAA AA |
| **CDK6** | F: | TGA TCA ACT AGG AAA AAT CTT GGA |
|  | R: | GGC AAC ATC TCT AGG CCA GT |
| **CCNA2** | F: | GGT ACT GAA GTC CGG GAA CC |
|  | R: | GAA GAT CCT TAA GGG GTG CAA |
| **CCNB1** | F: | ACA TGG TGC ACT TTC CTC CT |
|  | R: | AGG TAA TGT TGT AGA GTT GGT GTC C |
| **CCNB2** | F: | TGG AAA AGT TGG CTC CAA AG |
|  | R: | TCA GAA AAA GCT TGG CAG AGA |
| **CCND1** | F: | GCT GTG CAT CTA CAC CGA CA |
|  | R: | TTG AGC TTG TTC ACC AGG AG |
| **CCND2** | F: | GGA CAT CCA ACC CTA CAT GC |
|  | R: | CGC ACT TCT GTT CCT CAC AG |
| **CCNE1** | F: | GGC CAA AAT CGA CAG GAC |
|  | R: | GGG TCT GCA CAG ACT GCA T |
| **CCNE2** | F: | GCC ATT GAT TCA TTA GAG TTC CA |
|  | R: | CTG TCC CAC TCC AAA CCT G |
| **SMN-FL** | F: | CCT CCC ATA TGT CCA GAT TCT CTT GA |
|  | R: | TCT TTT TGA TTT TGT CTG AAA CCC ATA |
| **SMN-Δ7** | F: | CCT CCC ATA TGT CCA GAT TCT CTT GA |
|  | R: | TGC TCT ATG CCA GCA TTT CCA TAT |
| **SOX10** | F: | ACT TCG GCA ACG TGG ACA TT |
|  | R: | CAG CCA CAT CAA AGG TCT CCA T |
| **BRN3A** | F: | ACT TTA ACT TGC CCT TTC AG |
|  | R: | CTC TCC TAA TAA CTT TCA CCC |
| **TLX3** | F: | GCG CAT CGG CCA CCC CTA CCA GA |
|  | R: | CCG CTC CGC CTC CCG CTC CTC |
| **OCT4** | F: | GGA GAG CAA CTC CGA TGG |
|  | R: | TTG ATG TCC TGG GAC TCC TC |
| **NANOG** | F: | ATG CCT CAC ACG GAG ACT GT |
|  | R: | AGG GCT GTC CTG AAT AAG CA |
| **SOX1** | F: | GCG GAA AGC GTT TTC TTG |
|  | R: | TAA TCT GAC TTC TCC TCC C |
| **OLIG2** | F: | CAA ATC TAA TTC ACA TTC GG AAG GTT G |
|  | R: | CAA ATC TAA TTC ACA TTC GGA AGG TTG |
| **CHX10** | F: | GAA GAA GCG GCG ACA CAG |
|  | R: | GTG GGC TTC GTT GAA TGC |
| **FOXP1** | F: | GCA GTT ACA GCA GCA GCA CCT CC |
|  | R: | CAG CCT GGC CAC TTG CAT ACA CC |
| **SIM1** | F: | CTG TGG CGG CTA CAA GGT |
|  | R: | AGG CTG TAC TGG CGG ATC T |
| **PAX2** | F: | AAC GAC AGA ACC CGA CTA TG |
|  | R: | ATC CCA CTG GGT CAT TGG AG |
| **LHX1** | F: | AAG CCC ACA CGC CATATC CG |
|  | R: | GCT GTT TCA TCC TTC GCT CCT TG |
| **GDF11** | F: | ACC ACC GAG ACC GTC ATT AG |
|  | R: | AGG GCT GCC ATC TGT CTG |
| **HOXA6** | F: | GCA GCG GAT GAA CTC CTG |
|  | R: | GGT TGA AGT GGA ACT CCT TCT C |
| **HOXC8** | F: | CAT GTT TCC ATG GAT GAG ACC |
|  | R: | GGT CTG ATA CCG GCT GTA AGT T |
| **HOXC10** | F: | AGG AGA GGG CCA AAG CTG |
|  | R: | GCC AAT TTC CTG TGG TGT TT |
| **ChAT** | F: | GAC GTC TGA CGG GAG GAG |
|  | R: | TCA ATC ATG TCC AGC GAG TC |
| **ISL1** | F: | AAG GAC AAG AAG CGA AGC AT |
|  | R: | TTC CTG TCA TCC CCT GGA TA |
| **ACTB** | F: | CCA ACC GCG AGA AGA TGA |
|  | R: | CCA GAG GCG TAC AGG GAT AG |
| **GAPDH** | F: | AGC CAC ATC GCT CAG ACA C |
|  | R: | GCC CAA TAC GAC CAA ATC C |
